# Male Behavior Toward Reproductive Responsibilities in Sikkim

**DOI:** 10.4103/0970-0218.62552

**Published:** 2010-01

**Authors:** Yalley Dolma Chankapa, Ranabir Pal, Dechenla Tsering

**Affiliations:** Department of Health Care, Human Service and Family Welfare, Government of Sikkim, Sikkim, India; 1 Department of Community Medicine, Sikkim Manipal Institute of Medical Sciences (SMIMS) and Central Referral Hospital (CRH), 5^th^ Mile, Tadong, Gangtok, Sikkim-737 102, India

**Keywords:** Contraceptive, knowledge, practice

## Abstract

**Background:**

Failure to assess the impact of men's perceptions on reproductive health decisions has weakened reproductive health care programmes.

**Objectives:**

We evaluated husbands’ knowledge and practices with regard to the use of conventional contraceptives as manifested through reproductive health and sexual decisions.

**Materials and Methods:**

A population-based cross-sectional study was conducted in a rural setting of Sang PHC and Pakyong PHC area in Sikkim, India. Five hundred and ninety-six currently married men whose names were included in the eligible couple registers were selected by multistage random sampling. Information regarding knowledge and practice of contraceptive use was obtained from the participants by interview.

**Results:**

Out of the 596 male participants, the majority (55.87%) opined that they were in favor of using a contraceptive method after one child. Most participants (55.54%) said that their main source of information on contraceptive methods were the government health staff, while 24.84% acknowledged that most of their information came from the mass media. Eighty-two percent reported currently using some kind of the contraceptive method. Condom was used as a temporary method by only 16.27% of the responders, with the permanent method of vasectomy being opted for by only 4.87%. The method most widely used by their partners was the oral contraceptive pill (43.41%), followed by tubectomy (15.77%) and IUD (4.19%).

**Conclusions:**

This research found that awareness and prevalence of contraceptive use among married men in a rural community in the East District of Sikkim were quite high. Nevertheless, female contraceptive methods continue to be the dominant method used in the community. Researchers and health care providers often ignore the sociodemographic significance of men and their role in the acceptance of contraceptive practices in the community.

## Research Question

What was the level of knowledge and practice regarding contraceptive use among adult males in Sikkim?

## Introduction

Men play an essential role in reproduction. They should be encouraged to involve themselves in birth control, particularly in developing countries, where contraceptive usage goals have not yet been reached.(1) Reproduction calls for commitment from both partners but all too often in much of the world it is seen as wholly being the woman's responsibility. Failure to target men in reproductive health interventions has weakened the impact of reproductive health care programmes. Men's perceptions, as well as the determinants of sexual behavioral change and the socioeconomic context, should be reviewed. There is a need to study and foster change to reduce or prevent poor reproductive health outcomes and to identify behaviors which could be adversely affecting women's reproductive health. Issues of gender, identity, and tolerance as expressed through sexuality and procreation need to be followed up by positive reproductive health-promoting behaviors. Preventive reproductive health initiatives and information should move from focusing on the female alone and target both sexes. Women need to have men as partners in reproductive health—partners who understand the risks they might be exposed to and strategies for their prevention.(2)

Brazilian couples, in general, were not aware of any effective contraceptive options for use by men and/or participating in their use, except for vasectomy. The few methods with male participation that they knew of were perceived as methods that interfered in the spontaneity and pleasure of intercourse. Men accepted that condom use in extra-conjugal relations offered them protection from sexually transmitted diseases; that their wives might also participate in extramarital relationships was not considered. The paucity of contraceptive options that require male participation is one reason for the difficulty in sharing responsibilities between men and women. On the basis of perceived gender roles, women take on the responsibility for contraception until the moment when the situation becomes untenable, and they are faced with the unavoidable necessity of sterilization. Specific actions are necessary to achieve the participation of men in issues related to reproductive sexual health. These include education and discussions on gender roles, which should lead to greater awareness in men of the realities of sexual and reproductive health.(3)

In a study in the Turkey it was found that male university students who are sexually active generally do not have enough knowledge about family planning methods. They tend to engage in high-risk behavior. It is imperative that education and counseling in reproductive and sexual health be offered to all young men. In addition, men's attitudes toward contraceptive methods should be evaluated in other cultures and useful comparisons made with Turkey.(4) A study done in Bangladesh found association between men's reproductive health knowledge, attitude and behavior and their wives’ subsequent reproductive behavior; there was also significant association between husbands’ fertility preferences and current use of any family planning method.(5)

We evaluated husbands’ knowledge and practices with regard to conventional contraceptives as manifested through reproductive health and sexual decisions. So far there had been no study done in this field in the state of Sikkim in India.

### Objective

To determine husbands’ knowledge and practices with regard to contraceptive practices.

## Materials and Methods

A population-based cross-sectional study was conducted in a rural setting in Sikkim, India, from October 2003 to March 2004 (6 months). The areas covered by the Sang Primary Health Center (PHC) and the Pakyong PHC were selected for the study. Out of the four districts in Sikkim, namely East (Gangtok), West (Gyalshing), South (Namchi), and North (Mangan), the East District was selected randomly. In the East District, there are eight PHCs. Each PHC in Sikkim caters to a population of around 7000-15000.

First, permission from the office of the Principal Director was sought as the study involved subcenters. The Chief Medical Officer and the other PHC medical officers were requested by the office of the Health Directorate to cooperate with the investigators conducting the study. Then, two PHCs under the East District were selected randomly (by lottery method) in the office of the Chief Medical Officer, East District. The selected PHCs were Sang and Pakyong PHCs. In the third stage, from each of the selected PHCs, three subcenters were selected randomly (by lottery method). The selected subcenters were Ranka, Rumtek, and Martam (which are under Sang PHC) and Assam Lingzey, Aaho, and Changay Sente (which are under Pakyong PHC). In the fourth stage, 100 men were selected from each subcenter using the random number table. Thus, the total required number of 600 men were obtained. During the survey we could not get information from four of the selected participants as they could not be located even after repeated visits. These four were excluded from the study and we therefore had a total of 596 respondents from a reference population of 53,174 (Sang PHC - 24,586 and Pakyong PHC - 28,588).

The investigator visited each subcenter and arranged for the meetings with the male health workers (MHWs) posted there. Full cooperation from them was ensured after explaining the study in detail. The main point stressed was that the study was exclusively for the research purpose and was not intended as any kind of evaluation of their performance; they were also assured that full confidentiality would be maintained within the limits of medical ethics. All the eligible couples (EC) registers maintained in the subcenter were checked and a list was prepared of the men whose names were entered therein. All relevant information necessary for the study was noted down. For each center, a separate list was prepared from the EC register with the help of the MHWs of the particular subcenter. A rough guide map showing the locations of the selected households was prepared with the help of the Panchayat staff and the MHWs for quick identification of the participants and for prevention of duplication. All the participants were given explanations about the purpose of the study and were assured that strict confidentiality would be maintained. Verbal informed consent was taken from each of them before the interview. Data was collected by interview technique by the principal investigator. The collection of the data was done from 15^th^ of January to 30^th^ of March 2004. On an average, the principal investigator interviewed 7-8 men per day. Concurrently with the interview health education sessions were held to disseminate information on male contraception.

The data analysis involved transcription, preliminary data inspection, content analysis, and interpretation; we calculated the proportions and used the Chi-square test for statistical analysis.

### Content validity and reliability of the study instrument

The pre-tested close-ended questionnaire contained questions relating to the knowledge, opinion, and practices of husbands with regard to the contraceptive practices prevailing in India. The first part of the questionnaire focused on the sociodemographic and sociocultural characteristics, and the second part contained the questions related to the knowledge of contraceptive methods, source of information, opinion regarding ideal number of children, practice of contraceptive methods, etc. By initial translation, back-translation, and re-translation, followed by a pilot study, the questionnaire was customized for the study. The module was developed as anonymous interview schedule developed for the study in the Department of Health and Family Welfare, Government of Sikkim, with the assistance of my guide in Sikkim and other public health experts. For the pilot study, around 20 men from an another sub center adjacent to one of those sub centers selected for the study’ subcenter who fulfilled all the inclusion criteria, were administered the interview schedule by the principal investigator. Some of the questions from the interview schedule were modified till the investigator was satisfied with the response.

## Results

A total of 596 married men whose names were entered in the eligible couple register were interviewed. The minimum age of the men in the study was 19 years and the maximum was 56 years. The average number of children per couple was found to be 2.23 (range 0-7) [Table 1].

**Table 1:** Distribution of study population according to sociodemographic characteristics *(n* = 596)

Characteristics	Mean (± 2 SD)	Range
Age of respondent	32.9 (5.4)	19-56 years
Age of spouse	28.58 (6.8)	15-43 years
Total number of children	2.23 (1.1)	0-7
Number of sons	1.5 (0.8)	1-5
Number of daughters	1.5 (0.71)	1-6
Duration of marriage	9.43 (5.8)	1-26 years

Among the 596 subjects, 12.92% were illiterate; among the literate subjects, 317 (53.19%) had not completed primary school. With regard to occupation, 32.39% of the respondents were in government service, while 36.41% were manual laborers. When religion was considered, we found that the majority (70.31%) were Hindus, followed by Buddhists (24.00%). The majority (48.16%) belonged to the ‘other backward classes’ (OBC), and tribals comprised 31.38%. The time required for the men to reach the nearest health center was also assessed; 41.28% reported that it took them more than 1 h to reach the nearest health facility [Table 2].

**Table 2:** Distribution of the Government of Sikkim, sociocultural characteristics (*n* = 596)

Characteristics	Number	Percentage
Education		
Illiterate	77	12.92
Below primary school	317	53.19
Primary school to below higher secondary school	159	26.68
Higher secondary school and above	43	7.21
Occupation		
Government service	193	32.39
Private service	74	12.42
Cultivator	17	2.86
Manual labor	217	36.41
Business, artisans, unemployed and others	95	15.94
Religion		
Hindu	419	70.31
Buddhist	143	24.00
Christian	34	5.71
Ethnicity		
Schedule caste	37	6.21
Schedule tribe	187	31.38
OBCs	287	48.16
Others	85	14.27
Travel time to nearest health center		
Less than ½ hour	193	32.38
Half to 1 hour	157	26.34
More than 1 hour	246	41.28

Most of the respondents (55.54) said that their main source of information regarding contraceptive methods were government health staff; 24.84% said that their source was the mass media; and the rest reported friends, wife, or relatives as the primary source of information. [Table 3].

**Table 3:** Distribution of men according to source of information about various contraceptive methods *(n* = 596)

Source of information	Number of men *(n* = 296)	Percent
Health staff	331	55.54
Friends	57	9.57
Wives	13	2.19
Relatives	47	7.89
Media	148	24.84
Total	596	100

The majority (81.04%) of the men in our study opined that the ideal number of children to have was two, and among them the majority (55.87%) said that they would use a contraceptive method after one child; 33.23% preferred to use a contraceptive method after two children. In the matrix analysis of the timing of contraceptive use with the ideal number of children preferred, the preference of contraceptive use was noted to be significantly higher after the first child (Chi-square 70.813, df: 3, *P* < 0.0001). With regard to the ideal number of children, a significantly greater number of respondents stated a preference for a two-child limit (Chi-square 91.383, df: 3, *P* < 0.0001) [Table 4].

**Table 4:** Distribution of the study population according to timing of contraceptive use and opinion regarding ideal number of children (*n* = 596)

Timing of contraceptive use	Number of children preferred									

	One		Two		Three		Four		Total	
				
	Number	Percent	Number	Percent	Number	Percent	Number	Percent	Number	Percent
Immediately after marriage	22	78.57	6	21.43	0	0	0	0	28	4.70
After one child	7	2.21	283	89.27	17	5.36	10	3.15	317	53.19
After two children	2	1.01	132	66.66	42	21.21	22	11.11	198	33.22
After three children	0	0	28	62.22	11	24.44	6	13.33	45	7.55
After four and above	0	0	0	0	2	40.00	3	60.00	5	0.84
Non-response	0	0	0	0	1	33.33	2	66.66	3	0.51
Total	31	5.21	449	81.04	73	12.25	43	1.18	596	100

The current level of contraceptive use as reported by the men in the study was assessed for both male and female methods of contraception. Eighty-two percent of the respondents reported current use of some kind of contraceptive method. As the number of children increased, the rate of current contraceptive use also increased. But this trend was not significant (Chi-square for trend 0.1391, df: 1, *P* = 0.7092) [Table 5].

**Table 5:** Distribution of the study population according to information regarding current contraceptive use and timing of first contraceptive use (*n* = 596)

Timing of contraceptive use	Current contraceptive use					
	
	Total		Yes		No	
			
	Number	Percent	Number	Percent	Number	Percent
Immediately after marriage	28	4.37	20	71.43	8	28.57
After one child	317	55.87	273	86.12	44	13.88
After two children	198	33.23	163	82.32	35	17.68
After three children	45	6.21	31	68.89	14	31.11
After four and above	5	0.84	5	100	0	0
Did not respond	3	0.51	0	0	3	100
Total	596	100	492	82.55	104	17.45

When we analyzed the use of specific contraceptive methods, we found that the most widely used method by the female partner was the oral contraceptive pill (43.41%). The proportion of women in our study population using this method was significantly higher (*P* < 0.05) than those using other methods, viz, female sterilization or tubectomy (15.77%) and intrauterine devices (IUD) (4.19%). Among the males, condoms were used by 16.27%, while the permanent method of vasectomy was used by a very low percentage of men (4.87%). Overall, use of contraceptive methods by females was significantly higher than by males (*P* < 0.05) [Table 6].

**Table 6:** Distribution of methods of contraceptive use by specific method (*n* = 596)

Specific method	Number	Percent
Condom	97	16.27
IUD	25	4.19
Pills	247	43.41
Vasectomy	29	4.87
Tubectomy	94	15.77
Non user	104	17.45
Total	596	100

## Discussion

In this study, the prevalence of contraceptive use was 83.8%. The most commonly practiced method in the study was found to be oral pills, followed by tubectomy, condoms, and vasectomy. A survey conducted in Uttar Pradesh found the prevalence of use of contraceptives by males to be 40% compared to 25% reported by married women aged 13-49.(6) Some studies conducted on men have reported that men were aware of contraceptive methods but had little knowledge of their correct use.(7) It has been reported that the female contraceptive methods of oral pills was the most common method of contraception (41.6%) and was followed by female sterilization (16.6%) and condoms (16.5%). The least used methods were IUD (4.4%) and vasectomy (4.7%). In virtually all surveys of adult men, a large majority could identify at least one contraceptive method. In most of the countries Directorate of Health Services data on men, 90% or more of the men know of a contraceptive method.(8) In some of these countries all, or nearly all, men know of a contraceptive method. In this study, one variable that seemed to have an influence on contraceptive use was exposure to mass media. Studies have confirmed that after controlling for the other influences (like education), exposure to mass media has a substantial effect on contraceptive use.(9) Some studies have found that virtually all men (97%) know of at least one method of contraception, the information coming from the mass media.(10) Some studies have shown that a substantial number of married men know of at least one method of family planning, but only a small proportion of these actually practice contraception.(11)

While health care providers must be cautious not to attribute stereotypical religious, social, and cultural characteristics to women seeking advice about contraception, they do need to recognize that different value systems may influence contraception decision making in couples of different faiths. Along with this increased cultural awareness there is a need for understanding that each patient encounter is unique. The values that an individual woman holds may not be in keeping with the official teachings of her religion or the cultural norms reported by other members of the same culture.(12) In Turkey, contraceptive use was positively associated with both partners approving of family planning and negatively associated with both partners wanting more than three children and with only wives wanting three or fewer children. No evidence was found associating interspousal power differentials with method use.(13) In a study on the religious beliefs prevailing among Somali men living in Finland regarding the use of the condom by men and that of other forms of contraception by women found that 63% of the men avoided using condoms and were opposed to womens’ contraceptive use. The remaining 37% were not deterred by religious beliefs from using condoms or from approving of womens’ contraceptive practices. In brief, for religious reasons, most Somali men who were assessed avoided using condoms and disapproved of the use of contraception by women.(14)

In Tanzania, six themes emerged as overarching factors contributing to the vasectomy decision-making process: Economics, spousal influence, religion, provider reputation and availability, uncertainty about the future, and poor vasectomy knowledge and understanding. There was substantial communication between partners regarding the vasectomy decision, and wives had a strong influence on the outcome; however, men and women agreed that husbands would resist vasectomy if wives were to initially raise the topic. Vasectomy acceptance is limited by the scarcity of skilled vasectomy providers and by the fact that men and women hold many of the same misunderstandings about vasectomy, including a fear of decreased sexual performance as a result of the procedure. Spousal discussions are important in the decision to have a vasectomy, but these discussions should be initiated by the male partner.(15)

The results of this study indicate that despite increase in knowledge about contraceptives, female contraceptive methods continue to dominate among the contraceptive method mix.

### Strength of the study

Calls for increased involvement of men in matters of reproductive health emphasize the need for research into contraception acceptability and decision making. This research conducted into the attitudes and beliefs of Sikkimese men regarding contraceptive use attempts to explore the foundations of their sexual decisions. The principal investigator obtained all the responses personally after detailed probing, using the utmost care to avoid interviewer bias. Enough time and space was provided to each respondent for them to give their responses. Any doubts voiced by the respondent were cleared to their satisfaction without attempting to alter their original belief.

### Limitations of the study

Although the sample selection was done using the random number method, we considered only those married men whose names were entered in the eligible couples register. It is possible that the responses elicited could have been different if the study had included even those married men whose names were not included in the eligible couple register. A response bias can not be denied as we sought the help of the MHWs posted in the area to contact and locate the men from the selected households. Another limitation of the study was that the information available to measure the husband-wife communication was insufficient to measure the actual dimension and depth of communication. No information was collected concerning the duration, extent, and result of husband-wife communication on family planning. Another obvious limitation is one that is inherent to any study using cross-sectional data, i.e., the inability to make causal inferences.

## Conclusion

The findings of this research indicate that awareness and prevalence of contraceptive use among married men in this rural community in the East District of Sikkim was quite high, which is a very positive finding. Researchers and providers often ignore the social significance of men to define the factors that adversely affect reproductive health outcomes'.

### Recommendations

Researchers have reported various reasons cited by respondents for refusal to use contraceptives; for example, some men fear that if a woman is not at risk of pregnancy, she will be promiscuous. Other reasons for failure to use family planning methods include lack of communication between spouses, lack of access to contraceptives, the belief that women alone are responsible for fertility control, and the lack of availability of sufficient family planning information. Fear of side effects, desire to have more children, may be other reasons cited by married men for not using a contraceptive method. Making available a wide choice of methods at the community level should be a priority in family planning efforts. Given the findings in the present study, we make the following recommendations:

#### Behavior change communication

Health education is needed to improve knowledge about contraceptive use among married men with low educational status. There is a need for improving the educational tools, preferably with greater use of audiovisual techniques.

#### Dispelling myths and misconceptions

The information, education, and communication (IEC) system will have to focus on clearing the myths and misconceptions regarding conventional contraceptive methods and must keep the people well informed regarding the latest scientific advancements.

#### Provision of better facilities

The provision of better facilities could act as a motivating factor.
